# Transcranial sonography in carriers of Gaucher disease

**Published:** 2018-07-06

**Authors:** Fatemeh Omrani, Shahla Ansari-Damavandi, Babak Zamani, Zahra Omrani, Nahid Mohammadzade, Sadra Rohani, Mohammad Rohani

**Affiliations:** 1 Department of Neurology, Hazrat-e-Rasool Hospital, Iran University of Medical Sciences, Tehran, Iran; 2 Department of Pediatric Hematology, Ali Asghar Children's Hospital, Iran University of Medical Sciences, Tehran, Iran; 3 Department of Neurology, Firouzgar Hospital, Iran University of Medical Sciences, Tehran, Iran

**Keywords:** Transcranial Sonography, Gaucher Disease, Glucocerebrosidase, Parkinson's Disease, Substantia Nigra

## Abstract

**Background:** Glucocerebrosidase (GBA) mutation is the most common genetic risk factor in Parkinson’s disease (PD). Transcranial sonography (TCS) shows increased substantia nigra (SN) echogenicity in both idiopathic and genetic forms of PD. The goal of this study was to compare maximal area of SN hyperechogenicity (aSNmax) and diameter of third ventricle (DTV) between GBA mutation carriers and healthy controls.

**Methods:** Twenty-six carriers of GBA mutation and twenty-six healthy controls underwent TCS. The aSNmax and the DTV were measured. Mini-mental status examination (MMSE) and demographic data of the subjects were recorded, too.

**Results: **Mean aSNmax in GBA mutation carriers was significantly higher (0.31 ± 0.06 cm^2^) than controls (0.16 ± 0.04 cm^2^). Moreover, DTV was significantly higher in GBA mutation carriers group (3.98 ± 0.90 vs 3.29 ± 0.56 cm).

**Conclusion:** Increased SN echogenicity and increased third ventricle diameter in GBA mutation carriers may be caused by alterations in iron metabolism with reference to their genetic status.

## Introduction

Transcranial sonography (TCS) of brain parenchyma is a useful technique in evaluating movement disorders. About 90% of patients with idiopathic Parkinson’s disease (PD) show increased echogenicity of substantia nigra (SN).^1^^-^^3^ SN hyperechogenicity is also demonstrated in genetic forms of PD, including the ones with mutation in Parkin (PARK2), phosphatase and tensin homolog (PTEN)-induced putative kinase 1 (PINK1 or PARK6), leucine-rich repeat kinase 2 (LRRK2 or PARK8), and glucocerebrosidase (GBA).^1^^,^^2^ The degree of SN hyperechogenicity in genetic forms resembles those of sporadic ones.^1^^,^^2^ Heterozygous GBA mutations have been known as the most common genetic risk factor for PD.^4^^,^^5^ PD patients with this mutation clinically looks like patients without this mutation, except for earlier disease onset and more cognitive impairment in GBA mutation carriers.^4^ It should be mentioned that not all the GBA mutation carriers are neurologically symptomatic.^1^^,^^4^ On the other hand, non-manifesting carriers may show signs of PD later on; therefore, hyperechogenicity of SN can be a helpful marker in detecting preclinical phase of disease.^1^^,^^6^

Moreover, measuring diameter of the third ventricle (DTV) with TCS has emerged as a marker of brain atrophy and disease progression in PD.^7^

The goal of this study was to compare maximal area of SN hyperechogenicity (aSNmax) and third ventricle diameter between GBA carriers and healthy controls.

## Materials

Twenty-six GBA mutation carriers were collected from hematology department of a children’s hospital following ethical approval. We chose the carrier group from the parents of genetically-proved patients with Gaucher disease. Twenty-six healthy controls without neurological disease or a family history of PD were also recruited.

All the subjects were neurologically examined including mini-mental status examination (MMSE) and checking for the symptoms of PD (i.e. tremor, rigidity, and hypokinesia). Demographic data including age, sex, familial history of PD in first degree relatives, and smoking history (more than five pack years) were collected using a questionnaire. Both the case and control groups were given information about TCS prior to the test. The authors were committed to the declaration of Helsinki and all the patients signed consent forms.

**Figure 1:**
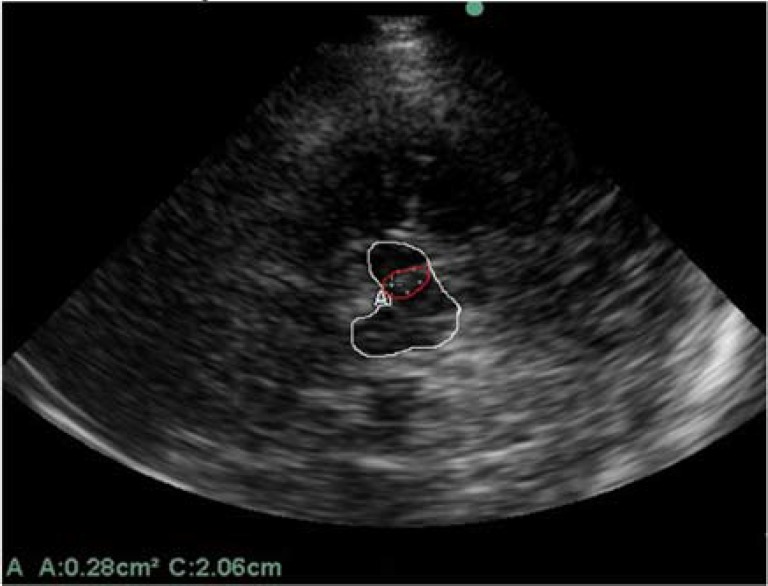
Transcranial sonography (TCS) of midbrain and maximal area of substantia nigra (SN) hyperechogenicity

All fifty-two subjects underwent TCS using the Sonos 5500 ultrasound system (Sonosite) equipped with a 2.0-2.5 MHz sector transducer (S3 probe). The sonographer was blinded to status of the subjects. TCS was done through right and left temporal bone windows with a penetration depth of 14-16 cm. SN echogenicity of each side was measured via ipsilateral temporal window (Figure 1). The larger SN area of each individual was selected for analysis (aSNmax). The distance between the inner margins of the third ventricle was considered as diameter of the third ventricle (DTV). 

We used SPSS software (version 20, IBM Corporation, Armonk, NY) for statistical analysis. For descriptive analysis, we measured mean and standard deviation, and for analytical part, we used parametric and non-parametric tests, independent sample t and one-way ANOVA tests.

## Results

The mean age of GBA mutation carriers was 35.57 ± 6.92 years, and the control was 34.92 ± 10.14 years.

The midbrain outline is colored in white and the SN area in red.

**Figure 2:**
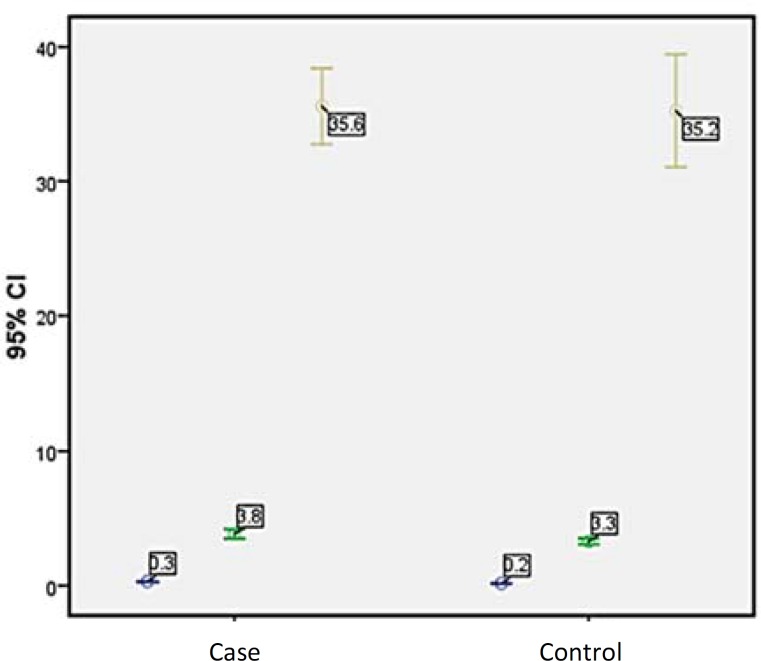
Error bar graph in case and control groups

The mean aSNmax and the mean diameter of third ventricle were significantly different between the two groups (Figure 2). Mean MMSE score was 29.80 ± 0.56 and 30.00 ± 0.00 in case and control groups, respectively (Table 1). 

The brown line stands for age, blue for diameter of third ventricle (DTV), and green for area of substantia nigra. CI: Confidence interval

19.23% of cases had a history of PD in their first-degree relatives, and 26.92% were smokers. According to non-parametric independent sample t test, aSNmax had statistical difference between the case and control groups (P < 0.001). Mean aSN max in control group was significantly lower than case group. Furthermore, third ventricle diameter was statistically higher in GBA mutation carriers (P = 0.013) (Table 1).

**Table 1:** Demographic and transcranial sonography of Glucocerebrosidase-mutation (GBA-mutation) carriers and healthy controls

**Feature **	**GBA mutation carriers ** **(mean ± SD)**	**Healthy controls ** **(mean ± SD)**	**P**
Age (year)	35.57 ± 6.92	34.92 ± 10.14	0.780
aSNmax (cm^2^)	0.31 ± 0.06	0.16 ± 0.04	< 0.001
DTV (cm)	3.83 ± 0.90	3.29 ± 0.56	0.013
MMSE	29.80 ± 0.56	30.00 ± 0.00	0.090

GBA: Glucocerbrosidase; aSNmax: Maximum area of substantia nigra hyperechogenicity; DTV: Diameter of third ventricle; MMSE: Mini-mental status examination; SD: Standard deviation

## Discussion

This study revealed that asymptomatic GBA-mutation carriers had greater SN echogenicity rather than healthy controls. This finding supports the idea that GBA mutation causes prominent dopaminergic cell loss in SN. Almost all autopsy proven cases of PD with GBA mutation had shown nigral cell loss.^4^^,^^8^ The etiology of Parkinsonism in GBA-mutation carriers is not yet fully understood. Probable mechanisms are reduced α-synuclein degradation, impaired lysosomal function, and lipid metabolism alteration leading to α-synuclein accumulation.^4^^,^^7^^,^^8^

In previous similar studies, such as Barrett, et al. study,^4^ GBA mutations were divided into mild and severe mutations based on heterozygous or homozygous subjects. Compared to mild GBA mutations, severe mutations were associated with an increased risk of developing PD and an earlier age of PD onset; but they did not find difference in SN echogenicity between cases with severe and mild mutations.^4^^,^^7^^,^^8^

Since we chose our cases from parents of patients with Gaucher disease, and did not check their genetic status, it was not possible to do such comparison. In addition, we did not assess genetically our control group which was another limitation of our study. The carrier frequency of Gaucher disease in the Ashkenazi Jews is approximately 6%, and is less than 1% in the non-Jewish population.^4^^,^^9^ Since none of our cases nor controls were Jews, we expect that this mutation rate is near to zero in our control group.

Diameter of the third ventricle is not broadly assessed with TCS in GBA-mutation carriers. In contrast to the study of Kresojevic, et al.^10^ our GBA-mutation carriers demonstrated significantly greater diameter than healthy controls. Since the third ventricle diameter is a marker of brain atrophy and neurodegeneration, GBA-mutation carriers can be prone to a neurodegenerative process; but there are many confounding factors, and we cannot confirm this hypothesis at this point.

In the study by Neumann, et al.^11^ no significant difference in aSNmax was found between asymptomatic GBA-mutation carriers and controls.

In the study of Saunders-Pullman, et al.,^12^ TCS demonstrated greater SN hyperechogenicity in GBA-mutation carriers than controls (0.28 vs. 0.14 cm^2^), which is compatible with our findings.

The increased SN echogenicity in GBA-mutation carriers may be due to alterations of midbrain iron metabolism without reference to their genetic status. In order to establish whether SN hyperechogenicity and increased DTV are linked to this mutation independent of PD, further studies with greater number of asymptomatic carriers are needed. Serial assessment of the SN echogenicity and autopsy pathological examinations in genetic and non-genetic forms of Parkinsonism may be helpful to recognize the causes of echogenicity changes.

## Conclusion

This study revealed increased echogenicity of SN and DTV in GBA-mutation carriers. Increased SN echogenicity in GBA-mutation carriers is similar to patients with idiopathic PD which supports the important role of GBA mutation in pathophysiology of PD.

## Notes:

**How to cite this article:** Omrani F, Ansari-Damavandi S, Zamani B, Omrani Z, Mohammadzade N, Rohani S, et al. Transcranial sonography in carriers of Gaucher disease. Iran J Neurol 2018; 17(3): 145-8.
